# Strongyloides Hyperinfection Syndrome Following Immunosuppressant Therapy for COVID‐19: A Case Report With Literature Review

**DOI:** 10.1002/ccr3.9689

**Published:** 2024-12-06

**Authors:** Robin Sharma, Muna Islam, Md. Kamrul Alam, Sudipta Das, Rabiul Islam, Aniruddha Ghose

**Affiliations:** ^1^ Chittagong Medical College Hospital Chattogram Bangladesh; ^2^ Bangabandhu Sheikh Mujib Medical University Dhaka Bangladesh

**Keywords:** Baricitinib, case report, corticosteroid, COVID‐19 pneumonia, immunosuppressant, Strongyloides hyperinfection syndrome

## Abstract

Strongyloides hyperinfection and disseminated infections are usually associated with immunosuppression; these severe manifestations occur in a minority of cases. The use of immunosuppressants such as corticosteroids and Baricitinib for treating COVID‐19 pneumonia can be responsible for patients' immunosuppression and cause Strongyloides hyperinfection syndrome. The chance increases when the patient belongs to countries or regions where chronic infection with Strongyloides is more prevalent. This case report describes the clinical scenario of a 78‐year‐old man from southeastern Bangladesh who was initially diagnosed with COVID‐19 pneumonia. His condition improved after receiving corticosteroid therapy for approximately 1 month at various doses and Baricitinib therapy for more than a week due to moderate‐to‐severe COVID‐19 pneumonia. Approximately 2 months later, he presented with low‐grade fever, diarrhea, and itching throughout the body. Blood analysis revealed eosinophilia; stool examination revealed Rhabditiform larvae of Strongyloides stercoralis. The patient was diagnosed with Strongyloides hyperinfection syndrome and treated with Albendazole and Ivermectin. His clinical condition gradually improved, and he was discharged from the hospital. The stool sample was sent for a repeat microscopic examination after 14 days, which yielded a negative result. Clinicians should be more vigilant while prescribing corticosteroids and other immunosuppressants for a prolonged period. Proper screening to identify asymptomatic cases of strongyloidiasis, followed by empirical treatment of screening‐positive cases, prompt detection, and management of severe manifestations, is crucial to reduce further morbidity and mortality related to Strongyloides stercoralis.

**Abbreviations**
ACE‐2: Angiotensin‐converting Enzyme ‐ 2
ARDS: Acute respiratory distress syndrome
COVID‐19: Coronavirus disease 2019
SARS‐CoV‐2: Severe acute respiratory syndrome coronavirus 2
WHO: World Health Organization

**Summary**

- Immunosuppression following corticosteroids or other immunosuppressants like Baricitinib can cause opportunistic infection, and Strongyloides hyperinfection syndrome can be one of them.
- Screening of patients from endemic countries worldwide and appropriate empirical treatment before immunosuppression are crucial to preventing hyperinfection syndrome. Early detection and management of severe manifestations are essential in mitigating further complications.

## 1 Introduction

Strongyloides stercoralis, a soil‐transmitted helminth mostly prevalent in tropical and subtropical regions with unsatisfactory sanitation systems, has been estimated to affect more than 600 million people worldwide [1, 2]. Among the WHO regions, Strongyloides is most prevalent in Southeast Asia [1]. There are several routes for transmission and multiple forms of disease manifestation, including acute, chronic, and hyperinfection syndrome [2]. Clinical manifestations of the infection include a wide range from being asymptomatic to various symptoms, hiding the real burden of the disease [2, 3]. Hyperinfection does not always result in disseminated strongyloidiasis, especially if it is correctly identified and treated; however, disseminated strongyloidiasis is a severe form of infection and, if not adequately addressed, is associated with a high mortality rate [4]. Immunosuppression following any condition or administration of immunosuppressive drugs can lead to hyperinfection and disseminated infection among chronically infected patients [3, 4]. As supported by several studies worldwide, using corticosteroids, independent of their dose and duration, is the most common trigger for Strongyloides hyperinfection and accelerating dissemination [3, 5, 6]. The use of corticosteroids for a short duration, such as 6 days or with a minimum dose of 20 mg/day of Prednisolone, has been reported to cause Strongyloides hyperinfection syndrome [2]. The concomitant use of other immunosuppressive agents with corticosteroids accelerates hyperinfection and dissemination, and in such cases, attributing a direct causal association with a specific agent becomes difficult [4].

Dexamethasone has been approved and commonly used in patients with moderate to severe COVID‐19 to prevent cytokine storms and the resulting ARDS, shock, and multiorgan failure, which can lead to death [7, 8]. Baricitinib, another drug approved for COVID‐19 treatment, is a reversible Janus kinase (JAK) inhibitor that can interrupt cytokine release during COVID‐19 and hinder viral entry into host cells by blocking the ACE2 receptor [9, 10]. Corticosteroids are strongly associated with Strongyloides hyperinfection, and Baricitinib, which has not yet been evidenced to be associated with hyperinfection, can still cause opportunistic infections [4, 9]. Therefore, the flare‐up of strongyloidiasis is a concern in such a situation. Several case studies of strongyloidiasis following COVID‐19 have been reported worldwide. However, to the best of our knowledge, no case of strongyloidiasis has been reported in Bangladesh.

As of September 30, 2,051,201 COVID‐19 cases have been confirmed in Bangladesh, and 29,499 deaths have been reported [11]. Corticosteroids, including Methylprednisolone, Dexamethasone, and Prednisolone, have been widely used in Bangladesh in the case of hospitalized COVID‐19 patients. The chances of strongyloidiasis following immunosuppression are greater among hospitalized COVID‐19 patients in highly prevalent regions like Bangladesh, where the estimated prevalence of Strongyloides stercoralis in 2017 was one of the highest, more than 15% [1]. In addition, having widespread symptoms and a resemblance to COVID‐19‐like manifestations [12] makes it difficult and can often cause delays in diagnosing and managing such patients. This case is being reported to highlight the suspicion, early detection, and prompt management of Strongyloides hyperinfection syndrome and prevent its further dissemination in countries like Bangladesh.

## 2 Case History

A 78‐year‐old man from Cox's Bazar (southeastern part of Bangladesh) was admitted to a hospital with complaints of fever and a productive cough for 3 days on April 10, 2021. His comorbidities included well‐controlled hypertension, ischemic heart disease, and benign prostatic enlargement (BPE).

A nasopharyngeal swab for SARS‐CoV‐2 came out positive on the day of admission. The patient's laboratory results just after admission showed a white blood cell count of 13.6 × 10^9^/L (reference range 4.00–11.00 × 10^9^/L), with 90% neutrophils (reference range 40%–75%) and 8% lymphocytes (reference range 20%–50%), and the C‐reactive protein level was 39.00 mg/L (reference range < 6.0 mg/L), D‐dimer 1.02 µg/mL (reference range < 0.5 µg/mL), and serum ferritin 301.50 ng/mL (reference range for male 18.2–341.2 ng/mL). High‐resolution chest computed tomography showed multiple bi‐basal ground glass opacities, and the patient gradually started to require oxygen supplementation. Treatment was commenced with intravenous Dexamethasone 6 mg/day, intravenous Remdesivir (100 mg) for 5 days, and oral Baricitinib (2 mg) for 10 days. The patient's oxygen requirement gradually decreased from 10 L/min via a face mask to maintaining oxygen saturation in room air. With this gradual improvement, he was discharged from the hospital 16 days after admission with a tapering dose of oral Dexamethasone for an additional 10 days.

The patient presented to the same hospital again 14 days after discharge, on April 30, 2021, with a high‐grade fever [39°C] and anorexia. History revealed that the fever was initially low‐grade and started just after the completion of oral Dexamethasone. On admission, his biochemical test results showed a white blood cell count of 11.2 × 10^9^/L with a neutrophil count of 70%, lymphocyte count of 8%, and eosinophil count of 2% (reference range 1%–6%). Additionally, his C‐reactive protein count was 126 mg/L, Serum ferritin was 637.40 ng/mL, and D‐dimer was 1.22 µg/mL. His oxygen saturation was also found to be fluctuating in room air and gradually required 2 to 4 L/min oxygen. Another nasopharyngeal swab for SARS‐CoV‐2 was done, and it turned out positive again. Oral Prednisolone was commenced this time for 2 weeks without antiviral and Baricitinib, considering COVID‐19 re‐infection. He gradually improved with this management and was discharged 7 days after the second admission.

The patient was admitted again for the third time to a different hospital, just 20 days after his second discharge (May 28, 2021), with loose stool, whole‐body itching, and low‐grade fever for approximately 3 to 4 days. He was treated there for loose stool, and then the patient was transferred to the previous hospital on June 2, 2021, where he had been admitted during his first two episodes of illness. This time, the patient had no oxygen demand.

## 3 Differential Diagnosis, Investigations, and Treatment

**FIGURE 1:**
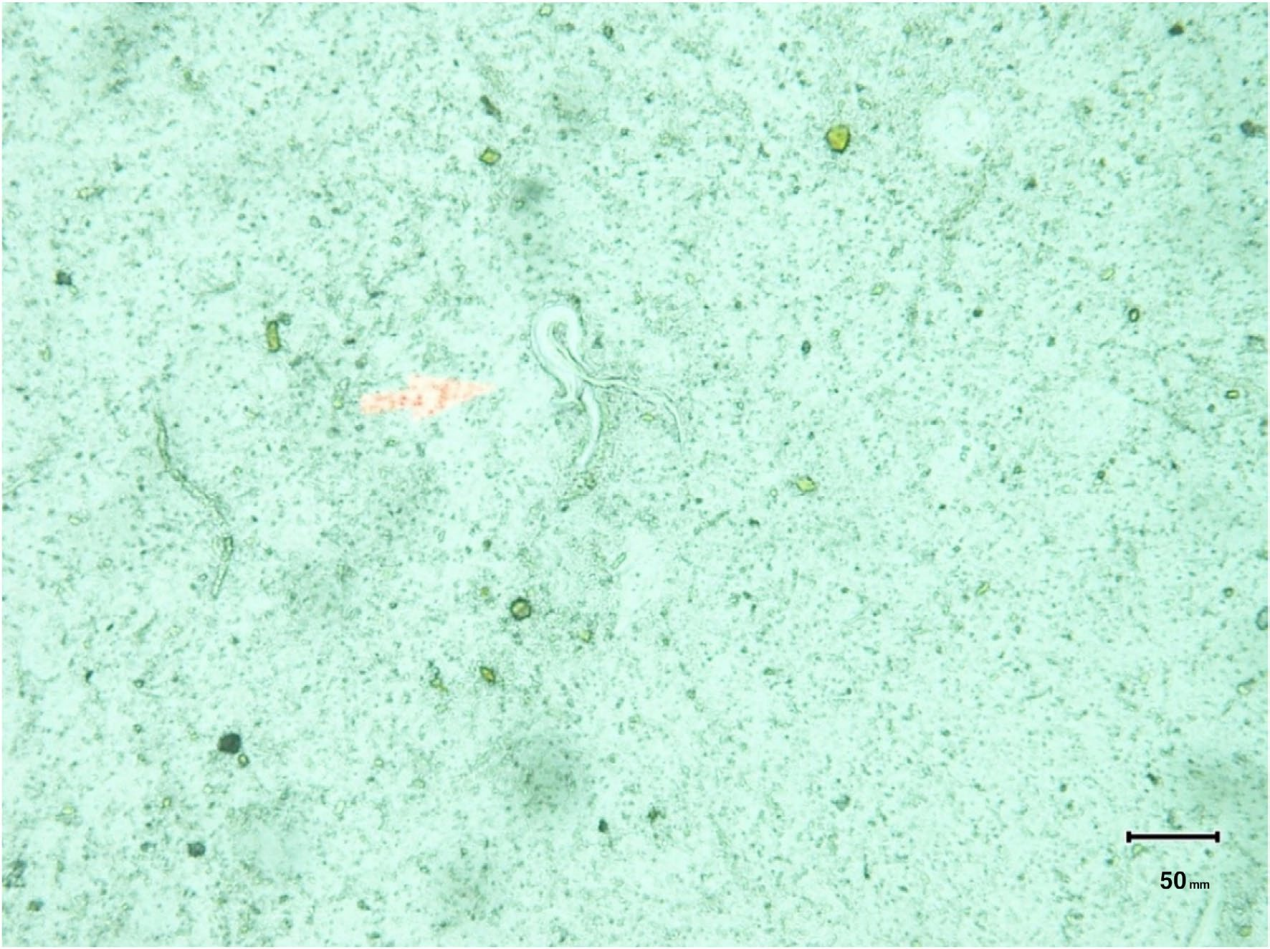
Stool routine microscopic examination showing Rhabditiform larva of Strongyloides stercoralis.

After admission, his blood tests revealed a white blood cell count of 26.5 × 10^9^/L with an eosinophil count of 49%. Differential diagnoses included Strongyloides hyperinfection syndrome and Ankylostomiasis. Stool routine microscopic examination was advised. After collecting stool samples, wet mount slides were prepared with normal saline and Lugol's iodine. These slides were reviewed by the microbiologist, which revealed the presence of Rhabditiform larvae of Strongyloides stercoralis in the stool (Figure 1). The Rhabditiform larvae were identified as having a short esophagus and a notched posterior end. The patient was then diagnosed with Strongyloides hyperinfection syndrome following immunosuppressant therapy due to COVID‐19. Treatment was commenced with oral Ivermectin 200 μg/kg for 14 days and oral Albendazole 400 mg on days 0 and 7.

## 4 Outcome and Follow‐Up

After 7 days of treatment, the patient became asymptomatic and was discharged from the hospital. A follow‐up stool sample taken after 14 days confirmed the absence of the parasite in the stool.

## 5 Discussion

Our patient was diagnosed during the first illness with moderate‐to‐severe COVID‐19 pneumonia and required supplemental oxygen, for which he was treated with intravenous corticosteroids and oral Baricitinib. Corticosteroids and Baricitinib were approved for patients with severe COVID‐19 to prevent cytokine storms [7, 8, 10]. However, using corticosteroids for a longer duration or at a greater dosage can cause immunosuppression, resulting in the reactivation of parasitic diseases; Strongyloides stercoralis hyperinfection is one such possibility, reported previously in multiple case reports worldwide [12, 13, 14]. Our patient had suffered from the same condition. Being immunosuppressed, associated with COVID‐19 treatment by corticosteroids and Baricitinib, and a resident of tropical and subtropical regions such as Bangladesh, he developed Strongyloides hyperinfection syndrome (SHS).

Several case studies worldwide reported severe manifestations of Strongyloidiasis (Strongyloides hyperinfection syndrome and disseminated strongyloidiasis) due to immunosuppression associated with COVID‐19 treatment [3, 12, 15, 16, 17, 18]. We have also found two case studies reported during the COVID‐19 pandemic that were associated with other immunosuppressive conditions, foretelling the chances of its flaring with immunosuppression following COVID‐19 [19, 20]. Of the 17 reported cases of Strongyloidiasis co‐infection with COVID‐19 pneumonia, approximately 95% were treated with corticosteroids. In two instances, Baricitinib was also used along with corticosteroids. Table 1 outlines the comparisons of the previously published Strongyloidiasis case reports in terms of immunosuppressants used as part of COVID‐19 treatment or other chronic conditions, the interval between COVID‐19 and strongyloidiasis symptoms onset, the clinical manifestations, investigation findings, treatment provided, and the patient's outcome.

**TABLE 1:** Comparative picture of the previously published cases of Strongyloidiasis (SHS & disseminated strongyloidiasis) during COVID‐19 pandemic^a^.

SI No.	Year of publication	Author [Ref. No.]	Age (Years), Sex (M/F), Country of origin	Immunosuppressant used [Duration/Dosage]	Interval between COVID‐19 and Strongyloidiasis symptoms onset	Chief complaints	Investigations Findings		Treatment provided	Outcome
							Blood	Other specimens		
1	2024	Setake [21]	91, M, Japan	1. Dexamethasone [Not Mentioned] 2. Baricitinib [Not Mentioned]	30 days	1. Abdominal pain 2. Vomiting 3. Loss of Appetite	1. No eosinophilia or eosinopenia	1. Sputum Microscopic Exam. (M/E)—Strongyloides sp. larvae 2. Stool M/E—Negative	Delayed diagnosis & treatment with Ivermectin	Deceased
2	2023	Busaidi [22]	55, M, Oman	1. Dexamethasone [6 mg/day for 5 days, and was prescribed 10 mg on a tapering dose for 5 weeks]	30 days	1. Diarrhea 2. Nausea 3. Loss of Appetite	1. Eosinophilia	1. Stool M/E—Rhabditiform larvae of Strongyloides sp.	Ivermectin & Albendazole	Improved
3	2023	Hamze [23]	64, M, Cuba	1. Dexamethasone [6 days] 2. Methotrexate (for Rheumatoid Arthritis)	Not specified	1. Diarrhea 2. Epigastric pain 3. Loss of Appetite	1. Eosinophilia 2. Strongyloides serology (ELISA)—Positive	1. Stool M/E—Positive	Ivermectin & Albendazole	Deceased
4^b^	2023	Soleymani [19]	67, W, Iran	1. Oral Prednisolone (10 mg) daily for Myasthenia Gravis	Not Applicable	1. Fever 2. Diarrhea 3. Vomiting 4. Weakness	1. Eosinophil count—Normal	1. Stool M/E—Positive for Rhabditiform larvae 2. Stomach tissue biopsy—Numerous eggs, Rhabditiform, and Filariform larvae	Ivermectin	Deceased
5	2022	Alkaabba [16]	76, M, USA	1. Dexamethasone [5 days]	14 days	1. Diarrhea 2. Abdominal pain 3. Vomiting 4. Nausea 5. Anorexia	1. Eosinophilia	1. Stool M/E—Positive	Ivermectin	Improved
6	2022	Babazadeh [24]	70, M, Iran	1. Dexamethasone [10 days]	21 days	1. Chest discomfort 2. Nausea 3. Loss of Appetite	1. Eosinophilia	1. Gastric and Duodenal Biopsy—Numerous eggs, Filariform larvae of Strongyloides sp.	Ivermectin & Albendazole	Improved
7	2022	Feria [25]	44, M, Bolivia	1. Dexamethasone [7 days]	7 days	1. Itching 2. New urticarial lesion on abdomen	1. Eosinopenia 2. Strongyloides serology (ELISA)—Positive	Not done	Ivermectin	Improved
8	2022	Feria [25]	74, F, Honduras	1. Dexamethasone [10 days]	10 days	1. Itching	1. Eosinopenia 2. Strongyloides serology (ELISA)—Positive	Not done	Ivermectin	Improved
9	2022	O'Dowling [26]	60, F, Nigeria	None (Asymptomatic COVID‐19)	Not determined	1. Abdominal pain	1. Strongyloides serology—Positive	1. Small bowel resection specimen—Presence of parasites of Strongyloides sp.	Ivermectin	Improved
10	2022	Kim [12]	63, M, Cambodia	1. Dexamethasone [10 days] 2. Baricitinib [5 days]	28 days	1. Fever 2. Worsening Respiratory Failure	1. Initially, Eosinopenia. then, Eosinophilia 2. Strongyloides Serum IgG—Positive	1. Stool M/E—Negative 2. Broncho Alveolar Lavage (BAL) fluid M/E—Parasites of Strongyloides sp.	Ivermectin	Deceased
11	2022	Singh [18]	58, M, India	1. Methylprednisolone [Duration not mentioned]	6 days	1. Abdominal pain 2. Vomiting 3. Itching	1. Eosinophilia	1. Stool M/E—Positive for Rhabditiform Larvae	Ivermectin & Albendazole	Improved
12	2021	Gautam [3]	53, M, India	1. Methylprednisolone [5 days]	60 days	1. Fever 2. Diarrhea 3. Abdominal discomfort	1. Eosinophil count—Normal	1. Stool M/E—Rhabditiform larvae 2. Stool Culture—Filariform larvae and adult female parasites	Ivermectin & Albendazole	Improved
13	2021	Marchese [27]	59, W, Italy	1. Dexamethasone [11 days] 2. Tocilizumab [2 doses]	25 days	1. Abdominal pain 2. Itching	1. Eosinophilia 2. IFAT serology—Positive	1. Stool M/E—Positive for Rhabditiform larvae	Ivermectin	Improved
14^b^	2021	Norman [20]	69, M, Colombia	1. Prednisolone 5 mg daily 2. Docetaxel, Atezolizumab, Ipatasertib (For stage IV prostate cancer with bone metastasis)	Not Applicable	1. Abdominal pain 2. Vomiting	1. Eosinophil count—Normal 2. Strongyloides serology (ELISA)—Positive	1. Stool M/E—Negative 2. BAL fluid M/E—Filariform larvae	Ivermectin	Improved
15	2021	Núñez‐Gómez [28]	45, M, Ecuador	1. Dexamethasone [Duration not mentioned]	12 days	1. Itching 2. Rash on trunk	1. Strongyloides serology screening—Positive	1. Stool Culture—Filariform larvae	Ivermectin	Improved
16	2021	Patel [15]	72, M, Nicaragua	1. Dexamethasone [Duration not mentioned]	Not mentioned	1. Persistent fever 2. Diarrhea	1. Eosinophilia	1. Stool M/E—Positive for Rhabditiform larvae 2. BAL gram stain—Larvae‐like body of Strongyloides sp.	Ivermectin	Improved
17	2021	Pintos‐Pascual [29]	70, M, Ecuador	1. Methylprednisolone [5 days] 2. Tocilizumab [8 days] 3. Anakinra [10 days]	55 days	1. Fever 2. Diarrhea 3. Epigastric pain 4. Vomiting 5. Nausea 6. Loss of appetite 7. Itching	1. Eosinophilia 2. Strongyloides serology—Positive	1. Stool M/E—Positive for Rhabditiform larvae	Ivermectin & Albendazole	Improved
18	2021	Stylemans [30]	59, M, Ecuador	1. Methylprednisolone [1 month] 2. Anakinra [Duration not mentioned]	60 days	Asymptomatic	1. Eosinophilia 2. Strongyloides serology—Positive	1. PCR of Stool sample—Positive for Strongyloides sp.	Ivermectin	Improved
19	2020	Lier [17]	68, M, Ecuador	1. Methylprednisolone [8 days] 2. Tocilizumab [1 dose]	18 days	1. Fever 2. Confusion	1. Eosinophilia	1. Sputum Culture–Positive for Rhabditiform larvae	Ivermectin & Albendazole	Improved

^a^ The cases are sorted based on the publication year.

^b^ Cases were reported during the COVID‐19 pandemic but were not associated directly with COVID‐19.

Based on previous case reports, it was evident that strongyloidiasis cases were more frequent among older adults and males; the same was noticed in this case, too. This patient, as in other cases, also received prolonged corticosteroids for more than a week and developed hyperinfection [12, 17, 19, 20, 22, 24, 25, 27, 30]. Baricitinib was also prescribed with corticosteroids to treat COVID‐19 in our patient, as in the previous two cases [12, 21]. Clinical manifestations of this patient included fever, diarrhea, and itching, and 6 out of 17, 7 out of 17, and 6 out of 17 previous cases reported the same symptoms, respectively [3, 12, 15, 16, 17, 18, 19, 22, 23, 25, 27, 28, 29]. This patient's initial complete blood count revealed leukocytosis and eosinophilia, aligning with the previous 11 cases [12, 15, 16, 17, 18, 22, 23, 24, 27, 29, 30]. Stool microscopy was advised upon suspicion, and Rhabditiform larvae were found; the previous nine cases reported the same [3, 15, 16, 18, 19, 22, 23, 27, 29]. Upon diagnosis, our patient was treated with an oral combination of Ivermectin and Albendazole. The previous seven cases of hyperinfection were treated with the same regime [3, 17, 18, 22, 23, 24, 29], while others were treated with Ivermectin alone [12, 15, 16, 19, 20, 25, 26, 27, 28, 30]. As did this patient, most of the previously reported cases survived and improved gradually with timely diagnosis and treatment.

According to Buonfrate et al., the countries with the loftiest prevalence of Strongyloides were mainly in the Southeast Asian, African, and Latin American regions [1]. Also, based on pooled Strongyloides seroprevalence mentioned in a previous study, migrants from these countries have a high prevalence of Strongyloides, reinforcing the need to take appropriate measures to avert Strongyloidiasis‐related complications associated with immunosuppression [31, 32].

To mitigate the mortality and morbidity due to complications related to strongyloidiasis, one possible strategy can be early diagnosis and treatment of complications such as Strongyloides hyperinfection syndrome. Delays in diagnosing hyperinfection syndrome and its timely management can lead to complications like disseminated infection and cause death, as happened in one previously reported case [21]. However, a lack of clinicians' awareness regarding the syndrome can be a contributing factor [31]. Another crucial strategy can be conducting Strongyloides screening before initiating immunosuppressant therapy of any duration, especially for patients hailing from the endemic zones [31, 33, 34]. Serological screening methods like IgG/IgM detection by enzyme‐linked immunosorbent assay (ELISA) and Strongyloides antigen detection by rapid immunochromatographic test (ICT) have previously been used as screening methods [35, 36]. Empirical Ivermectin therapy for screening‐positive strongyloidiasis patients before immunosuppression can be an effective strategy to mitigate further complications [37].

This case report was intended to describe the case of a Strongyloides hyperinfection resulting from corticosteroids and Baricitinib used to treat COVID‐19. Corticosteroids are proven immunosuppressants, tending to cause hyperinfection. However, two previous cases documented Baricitinib as an immunosuppressant, causing hyperinfection. Since, in both cases, Baricitinib was used with corticosteroids, it is difficult to ascertain if there is any causal relation between the use of this drug and the development of hyperinfection.

Bangladesh has a high prevalence of Strongyloides stercoralis. Yet, to the best of our knowledge, this is the first case report of Strongyloides hyperinfection syndrome associated with COVID‐19 infection being reported. Several disease conditions and medications, other than corticosteroids, can cause immunosuppression. So, this case study can make an excellent example for clinicians from highly Strongyloides‐prevalent areas, as well as clinicians from overseas, having migrants from prevalent countries, to rule out Strongyloidiasis through proper screening and treat if necessary to prevent further complications.

## 6 Conclusion

Clinicians should exercise a more cautious approach before commencing any form of immunosuppressive treatment, as the prevalence of chronic asymptomatic strongyloidiasis cases is not insignificant, particularly in the endemic zone. Screening for Strongyloides can be an effective tactic to identify asymptomatic strongyloidiasis cases, and empirical treatment in positive cases might avoid severe manifestations like Strongyloides hyperinfection syndrome and disseminated strongyloidiasis. If presented with features consistent with hyperinfection, prompt diagnosis, and treatment can prevent further progression to dissemination, eventually reducing morbidity and mortality associated with Strongyloides stercoralis.

## Author Contributions

**Robin Sharma:** conceptualization, data curation, formal analysis, investigation, methodology, supervision, validation, visualization, writing – original draft, writing – review and editing. **Muna Islam:** conceptualization, data curation, formal analysis, investigation, methodology, supervision, validation, visualization, writing – original draft, writing – review and editing. **Md. Kamrul Alam:** conceptualization, data curation, formal analysis, investigation, methodology, supervision, validation, visualization, writing – original draft, writing – review and editing. **Sudipta Das:** conceptualization, data curation, formal analysis, investigation, methodology, supervision, validation, visualization, writing – original draft, writing – review and editing. **Rabiul Islam:** conceptualization, data curation, formal analysis, investigation, methodology, supervision, validation, visualization, writing – original draft, writing – review and editing. **Aniruddha Ghose:** conceptualization, data curation, formal analysis, investigation, methodology, supervision, validation, visualization, writing – original draft, writing – review and editing.

## Ethics Statement

The authors have nothing to report.

## Consent

Written informed consent was obtained from the patient to publish the case report and any accompanying images.

## Conflicts of Interest

The authors declare no conflicts of interest.

## Data Availability

This published article includes all the data generated or analyzed during this study.
